# Fistula formation into other organs secondary to intraductal papillary mucinous neoplasm of the pancreas: A case report and literature review

**DOI:** 10.1097/MD.0000000000034288

**Published:** 2023-07-07

**Authors:** Yutaka Shishido, Eisei Mitsuoka, Rieko Ito, Masayuki Ishii, Koji Fujimoto

**Affiliations:** a Department of Gastrointestinal Surgery, Shinko Hospital, Kobe, Japan; b Department of Diagnostic Pathology, Shinko Hospital, Kobe, Japan.

**Keywords:** pancreas, intraductal papillary mucinous neoplasm, fistula, case report, literature review

## Abstract

Fistula formation from intraductal papillary mucinous neoplasm (IPMN) of the pancreas is uncommon; however, IPMN penetrating various organs has been increasingly reported. To date, there is a lack of literature reviewing recent reports and the clinicopathologic details of IPMN with fistula formation are still poorly understood.

**Methods:**

This study describes the case of a 60-year-old woman presenting with postprandial epigastric pain and diagnosed with main-duct (MD) IPMN penetrating to the duodenum, and presents comprehensive literature review of IPMN with fistulae. A literature review was performed using PubMed for all articles in English using predetermined search terms, including (fistula or fistulization), (pancreas or pancreatic or pancreato or pacreatico), (intraductal papillary mucinous), and (neoplasm or tumor or carcinoma or cancer).

**Results:**

A total of 83 cases and 119 organs were identified in 54 articles. Affected organs were as follows: the stomach (34%), duodenum (30%), bile duct (25%), colon (5%), small intestine (3%), spleen (2%), portal vein (1%), and chest wall (1%). Fistula formation into multiple organs was detected in 35% of cases. Approximately one-third of the cases had tumor invasion around the fistula. MD and mixed type IPMN accounted for 82% of cases. IPMN with high-grade dysplasia or invasive carcinoma were over three times more common than IPMN without these components.

**Discussion and conclusion:**

Based on the pathological examination of the surgical specimen, this case was diagnosed of MD-IPMN with invasive carcinoma and mechanical penetration or autodigestion was considered as the mechanism of fistula formation. Given the high risk of malignant transformation and intraductal dissemination of the tumor cells, aggressive surgical strategies, such as total pancreatectomy, should be recommended to achieve complete resection for MD-IPMN with fistula formation.

## 1. Introduction

Intraductal papillary mucinous neoplasm (IPMN) is a pancreatic exocrine tumor consisting of intraductal papillary growth of mucin-producing neoplastic cells within the pancreatic duct. IPMN accounts for less than 10% of all pancreatic neoplasms and exhibits a unique range of clinical features compared to invasive ductal adenocarcinoma.^[1]^ IPMNs display a broad spectrum of dysplasia, ranging from low and high-grade dysplasia (carcinoma in situ) to invasive carcinoma.^[2]^ Regarding its prognosis, IPMN with invasive carcinoma has less aggressive histopathological features and a better survival time than pancreatic ductal adenocarcinoma.^[3]^ IPMN is morphologically categorized into 3 types based on imaging studies and/or histology: main-duct (MD), branch-duct (BD), and mixed type.^[4]^ MD-IPMN is defined as IPMN with partial or diffuse dilatation of the main pancreatic duct exceeding 5 mm without other obstructive causes. BD-IPMN is characterized by the presence of pancreatic cysts larger than 5 mm communicating with the main pancreatic duct. Mixed type IPMN meets the criteria for both MD-PMN and BD-IPMN.^[4]^ MD-IPMN is more often associated with invasive carcinoma than BD-IPMN,^[5]^ while BD-IPMN has the risk of concomitant pancreatic ductal adenocarcinoma.^[6]^ Therefore, the morphological classification is crucial for determining the treatment strategy for IPMN.^[4]^

IPMN occasionally forms fistulas penetrating adjacent organs, such as the duodenum, stomach, and common bile duct.^[7]^ Additionally, despite the relatively favorable prognosis of IPMN, IPMN with fistula formation was reported to have a poor prognosis, which was compatible with pancreatic intraductal adenocarcinoma.^[3,7]^ Due to improved diagnostic and imaging techniques, reports of IPMN with fistula formation to various organs have been increasing in recent years. However, studies involving comprehensive literature review of such reports are limited. Moreover, the clinicopathological characteristics of this phenomenon remain unclear. Herein, we describe a case of IPMN of the pancreas penetrating to the duodenum with clear images. Furthermore, we present a literature review on fistula formation secondary to IPMN of the pancreas to gain a better understanding of its pathophysiology.

## 2. Case presentation

**Figure 1.**
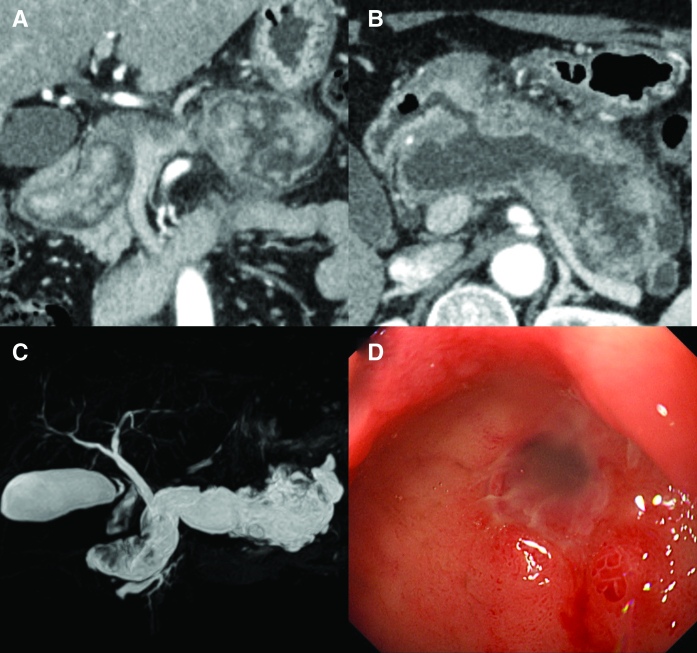
(A) Contrast-enhanced computed tomography of the abdomen reveals an enlarged pancreas with multiple papillary masses in the main pancreatic duct and extensive ductal dilation. (B) Computed tomography shows a fistula between the main pancreatic duct and the duodenum. (C) Magnetic resonance cholangiopancreatography shows an enlarged pancreas with extensive ductal dilation. (D) Esophagogastroduodenoscopy shows a fistula with mucus discharge in the duodenal bulb.

A 60-year-old woman presenting with postprandial epigastric pain and a deep duodenal ulcer detected on esophagogastroduodenoscopy was referred to our hospital for evaluation. She was not receiving any medication but had undergone surgery for right leg varicose veins 3 years ago. At presentation, there were no abnormal findings on physical or laboratory examinations, including tumor markers. An abdominal contrast enhanced computed tomography showed an enlarged pancreas with multiple papillary masses in the main pancreatic duct and extensive ductal dilation (maximum diameter: 40 mm) (Fig. 1A). The tumors were predominant in the head and tail of the pancreas. The tumor in the tail extended outside the pancreas, and invasion of the stomach was suspected.

Furthermore, a fistula between the main pancreatic duct and the duodenum was observed (Fig. 1B). Magnetic resonance cholangiography confirmed the dilatation of the main pancreatic duct initially observed on the contrast enhanced computed tomography (Fig. 1C) and showed multiple intraductal tumors with low signal intensity on the T2-weighted images. Esophagogastroduodenoscopy at our hospital showed a fistula with mucus discharge in the duodenal bulb (Fig. 1D). Brush cytology of the pancreatic duct demonstrated atypical glandular cells with papillary growths and mucus production. Based on these findings, MD-IPMN of the pancreas with a fistula penetrating the duodenum was strongly suspected.

**Figure 2.**
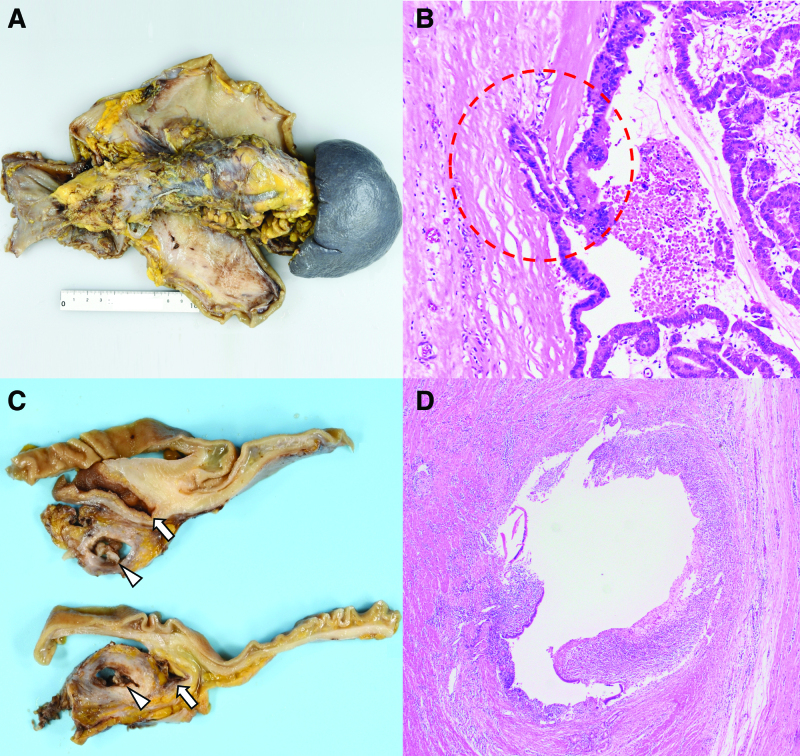
(A) Surgical specimen of the enlarged pancreas, stomach, duodenum, and spleen. (B) Tumor cells with papillary proliferation and nuclear atypia infiltrate the pancreatic duct wall (red dot-line) (Hematoxylin and eosin staining, magnification 4×). (C) Intraductal tumor (arrowheads) and fistula formation to the duodenum (arrows) are macroscopically identified. (D) The fistula comprised duodenal mucosal cells and granulation tissue with dense inflammatory cell infiltration (Hematoxylin and eosin staining, magnification 20×).

Total pancreatectomy, splenectomy, cholecystectomy and subtotal gastrectomy were performed to achieve en-bloc tumor resection (Fig. 2A), because of the extensive pancreatic lesion and firm adhesions between the gastric body and the pancreas. On pathological examination, the tumor was 150 mm in length and tumor cells with papillary proliferation and nuclear atypia infiltrated the pancreatic duct wall (Fig. 2B). Therefore, the patient was diagnosed with an IPMN of the pancreas with invasive carcinoma. Fistula formation to the duodenum was macroscopically identified (Fig. 2C). No malignant cells were observed in the fistula, which consisted of duodenal mucosal cells and granulation tissue with dense inflammatory cell infiltration (Fig. 2D). There was no direct invasion of tumor cells into the gastric wall. The patient’s recover was uneventful. She was discharged on regular subcutaneous insulin injections for glycemic control. Follow-up at 7 months postoperatively found no evidence of recurrence.

## 3. Discussion

**Figure 3.**
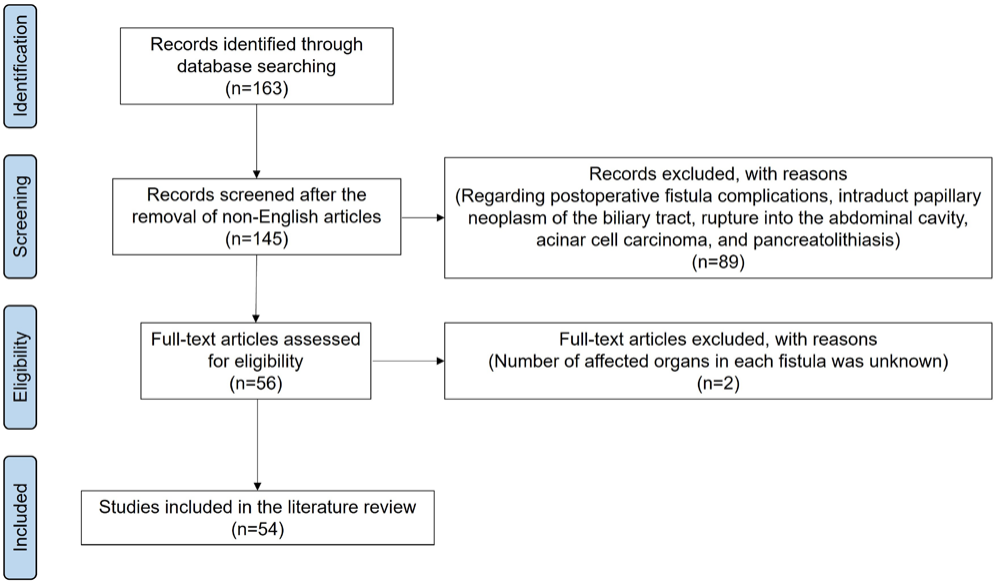
Flow diagram of the study selection process for the literature review.

Fistula formation into adjacent organs is a unique complication of IPMN, with a reported prevalence of 1.9% to 6.6% in patients with IPMN.^[7,8]^ With the improvement of endoscopic and imaging technology, IPMN penetrating various organs has been increasingly reported in recent years. However, the clinicopathologic details of IPMN with fistula formation are still poorly understood, and there is a lack of literature reviewing recent reports. Therefore, we comprehensively searched the PubMed databases for articles through February 15, 2023. The search terms included (fistula OR fistulization) AND (pancreas OR pancreatic OR pancreato OR pacreatico) AND (“intraductal papillary mucinous”) AND (neoplasm OR tumor OR carcinoma OR cancer). We included articles that reported fistula formation from IPMN of the pancreas and were published in English. We excluded articles in which the number of affected organs in each fistula was unavailable. Ultimately, we found 54 articles, including 3 retrospective studies,^[7–9]^ 36 case reports,^[10–46]^ and 15 brief imaging articles^[47–60]^ (Fig. 3). Fistula formation into other organs secondary to IPMN of the pancreas was reported in 83 cases and 119 organs. Table 1 summarizes the affected organs, case numbers, morphological type of IPMN, presence of malignant components including high-grade dysplasia and carcinoma, and tumor invasion around the fistula.

**Table 1:** Clinicopathological data of intraductal papillary mucinous neoplasm of the pancreas with fistula formation.

Affected organs	Case	Type		Malignant component			Invasion around fistula			References
		MD/mixed	BD	Yes	No	N/A	Yes	No	N/A	
Stomach	18	17	1	7	7	4	1	9	8	^[7,13,15,22,24,28,31,35,40,44,51–53,56–58]^
Duodenum	12	9	3	7	2	3	1	2	9	^[7,8,30,38,45,48,55]^
Bile duct	21	18	3	12	2	7	1	9	11	^[7,10,12,14,18,19,25,26,32,34,37,39,42,43,49,60]^
Small intestine	1	0	1	0	0	1	0	0	1	^[7]^
Colon	1	1	0	1	0	0	0	1	0	^[17]^
Portal vein	1	1	0	1	0	0	1	0	0	^[54]^
Stomach, duodenum	11	10	1	7	2	2	5	2	4	^[7,8,16,20,21,33,41,47]^
Stomach, bile duct	1	1	0	0	1	0	0	1	0	^[27]^
Stomach, colon	2	0	2	2	0	0	1	0	1	^[50,59]^
Stomach, spleen	1	1	0	1	0	0	0	1	0	^[36]^
Stomach, chest wall	1	1	0	1	0	0	1	0	0	^[11]^
Duodenum, bile Duct	5	2	3	2	0	3	1	0	4	^[7,8,23,34]^
Duodenum, colon	1	1	0	0	0	1	0	0	1	^[8]^
Duodenum, small intestine	1	1	0	1	0	0	0	1	0	^[9]^
Stomach, duodenum, bile duct	3	3	0	2	0	1	0	0	3	^[7,8]^
Stomach, duodenum, colon	1	0	1	1	0	0	1	0	0	^[7]^
Stomach, duodenum, small intestine	1	1	0	0	0	1	1	0	0	^[29]^
Stomach, duodenum, colon, spleen	1	1	0	1	0	0	0	0	1	^[46]^
Total number	83	68	15	46	14	23	14	26	43	

BD = branch-duct, MD = main-duct, N/A = not applicable.

To date, organs fistulated by IPMN include the stomach, duodenum, bile duct, small intestine, colon, portal vein, spleen, and chest wall, as shown in Table 1. Theoretically, IPMN could form fistulas in all adjacent organs. In our case, fistula formation by IPMN was detected in only the duodenum. Based on our literature review, the most frequently reported organs were as follows: the stomach (40/119 organs, 34%), the duodenum (36/119 organs, 30%), the bile duct (30/119 organs, 25%), and the colon (6/119 organs, 5%). Large contact areas with the pancreas could explain the higher incidence of fistula formation into the stomach, duodenum, and bile duct. Fistula formation into solid organs, such as the spleen, is rare compared to luminal organs due to the higher resistance of solid organs to increased intraductal pancreatic pressure, which is assumed to be one of the mechanisms for fistula development.^[36]^ Fistula formation into multiple organs was detected in 29 cases (29/83 cases, 35%), and the most frequent combination was the stomach and the duodenum. However, the interpretation of organ prevalence should be conducted with caution. This review included a lot of case reports, and publication bias was likely since rare cases are more likely to be reported.

The mechanism of fistula formation caused by IPMN remains controversial. Several theories regarding the pathogenesis of fistula formation have been proposed, such as direct invasion of tumor cells, mechanical penetration, and autodigestion by pancreatic enzymes.^[7,9]^ According to our review, direct invasion of IPMN was found in approximately one-third of the cases for which pathological information was available. Tumor invasion around the fistula is more common in cases with multiple organ fistulas, which might reflect the more aggressive features of IPMN with multiple organ fistulas. The other 2 mechanisms could have contributed to fistula formation in the remaining two-thirds of the cases. Mechanical penetration could be the result of increased intraductal pressure due to the high mucus production by IPMN and associated inflammatory changes. Autodigestion by pancreatic enzymes was also mentioned as one of the possible mechanisms based on the pathologically proven dissolution of the pancreatic duct wall and parenchyma.^[9]^ Furthermore, the combined effects of these 2 mechanisms were suggested by Yamada et al^[9]^ as follows: increased intraductal pressure erodes through the pancreatic duct epithelium; autodigestion causes dissolution of the pancreatic duct and parenchyma, and perforation of the pancreatic duct into the peripancreatic fat tissue; inflammation of the peripancreatic fat results in adhesions between the pancreatic duct and adjacent organs; autodigestion of adjacent organs eventually forms fistulae. In our case, there was no pathological evidence of tumor invasion around the fistula, suggesting mechanical penetration or autodigestion as the underlying cause of fistula formation.

IPMN is known as a slow-growing tumor with a relatively good prognosis. A meta-analysis showed that IPMN with invasive cancer had a better median survival time of 21–58 months compared to 12–23 months for pancreatic ductal adenocarcinoma.^[3]^ However, IPMN with fistulae was reported to have a poorer prognosis, with a median survival of 16 months.^[7]^ If tumor cells are present around the fistula, the lesion could be considered direct invasion to other organs and associated with a higher T stage (T3-4) and worse prognosis.^[61]^ Regarding the classification of IPMN, our study showed that MD-IPMN and mixed type IPMN accounted for the majority (82%) of cases of IPMN with fistula formation. The malignancy rate of BD-IPMN, MD-IPMN, and mixed-type IPMN was reported to be 24.4%, 62.2%, and 57.6%, respectively.^[5]^ As shown in Table 1, IPMNs with malignant components were over three times more common than IPMNs without malignant components. The prognosis of IPMN with invasive cancer is significantly worse than without invasive components.^[62]^ Therefore, the number of MD-IPMN and mixed type IPMN in the fistula-forming cases could correlate with its poor prognosis. Furthermore, MD-IPMN is characterized by the development of multiple monoclonal lesions due to intraductal dissemination.^[63]^ Hence, MD-IPMN with fistulae has the potential for tumor cells to disseminate to multiple organs through the fistulae. The presence of tumor cells in the biliary duct was confirmed pathologically in the case of IPMN with a pancreato-biliary fistula.^[18]^

Due to its rarity and broad spectrum of dysplasia, the treatment strategy for IPMN with fistula has not been established. In the case of MD-IPMN, extended pancreatic resection, such as pancreatoduodenectomy, could be indicated to achieve a negative surgical margin, considering the high risk of malignant transformation and intraductal dissemination of the tumor cells.^[5,63]^ Furthermore, even if there were no tumor cells around the fistula, tumor dissemination to the affected organs could still happen via the fistula.^[18]^ Therefore, aggressive surgical strategies, such as extended pancreatectomy and simultaneous resection of infiltrated organs, should be recommended in patients with MD-IPMN with fistula formation. However, in cases with BD-IPMN, more conservative treatment is recommended, given the lower malignant potential.^[5]^ Moreover, fistula formation from BD-IPMN should not always necessitate surgery, considering the possibility of nonmalignant mechanisms.^[8]^ Therefore, resection of the fistula is not required if preoperative examination could demonstrate the absence of malignant cells around the fistula. Hence, active monitoring without surgery might be indicated for BD-IPMN with fistula formation, considering the risk of concomitant pancreatic ductal adenocarcinoma.^[6]^ In our patient, total pancreatectomy, cholecystectomy, splenectomy, and subtotal gastrectomy were performed to achieve en-bloc tumor resection given the extensive pancreatic lesion and aggressive characteristics of MD-IPMN with fistula formation and the suspicion of direct invasion to the stomach.

## 4. Conclusion

We have experienced an uncommon case of fistula formation from IPMN into the duodenum. Based on the pathological examination of the surgical specimen, a definitive diagnosis of MD-IPMN with invasive carcinoma was made, and mechanical penetration or autodigestion was considered as the mechanism of fistula formation. Given its unfavorable characteristics and poor prognosis, aggressive surgical strategies should be recommended to achieve complete resection for MD-IPMN with fistula formation.

## Author contributions

**Conceptualization:** Yutaka Shishido.

**Data curation:** Yutaka Shishido.

**Formal analysis:** Yutaka Shishido.

**Investigation:** Yutaka Shishido.

**Project administration:** Yutaka Shishido.

**Resources:** Yutaka Shishido, Eisei Mitsuoka, Rieko Ito.

**Writing – original draft:** Yutaka Shishido.

**Writing – review & editing:** Yutaka Shishido, Eisei Mitsuoka, Rieko Ito, Masayuki Ishii, Koji Fujimoto.
